# Assessment of Recidivism Risk in Sex Offenders: A Pilot Study in Central Italy

**DOI:** 10.3390/healthcare9111590

**Published:** 2021-11-20

**Authors:** Barbara Gualco, Franco Scarpa, Regina Rensi

**Affiliations:** 1 Section of Medical Forensic Sciences, Department of Health Sciences, University of Florence, 50100 Florence, Italy; barbara.gualco@unifi.it; 2 Forensic Mental Health’s Service, USL Center Tuscany, 50100 Firenze, Italy; franco.scarpa@uslcentro.toscana.it

**Keywords:** sex offenders, recidivism, assessment

## Abstract

Knowing the risk factors of recidivism in sex offenders is important in order to prepare effective preventative interventions and treatment in custody. In this regard, the following paper shows the results of a pilot study carried out in the prisons of central Italy in which 44 sex offenders participated. These participants were given the following tests: Historical Clinical Risk Management-20-version 3 (HCR20v3), Psychopathy Checklist-Revised (PCL-R) and Personal Inventory Dimensional (PID-5). The results show a high positivity in the factors of the sub-scales H (historical factors) and C (clinical factors) of HCR20v3; the average total score of the PCR-L is 16.47, with five subjects who are in the “high psychopathy” range (X ≥ 30); with regard to PID-5, the most positive domain is negative affectivity (56.10%).

## 1. Introduction

Sexual violence is a public health problem with serious and long-term effects for the offender and the victim, defined by the World Health Organization [1] as “any sexual act directed against a person without his consent and through the use of coercion”. For this reason, there are numerous studies that have tried to discern which risk factors and personality traits can contribute to a person becoming a sex offender [2,3,4,5]. Some studies have focused on the cognitive distortions [6,7,8,9,10,11,12] that sex offenders have towards women, children, and the world in general. Examples of cognitive distortions most prevalent in sexual abusers include the following: not being able to control one’s own behavior; the perception of the world as dangerous, which leads to seeing the other as threatening and justifying abuse; considering women and children to be sexual objects; and denigration, denial, and rejection of the pain/damage caused to the victim [13]. These beliefs allow the offender to justify their deviant behavior, and reduce the perceived discomfort and the sense of guilt and shame attached to the unacceptable behavior. This puts the thought in the offender’s mind that his behavior can be repeated [14,15]. In addition to distorted belief systems, sex offenders share other crime-linked characteristics such as poor ability to achieve a dimension of intimacy, the feeling of a strong sense of loneliness and difficulty in social relationships, poor coping strategies, and low self-esteem [5,16,17,18]. Another factor that sexual assailants seem to share is a lack of empathy, a complex emotion that requires articulated metacognitions in order for it to develop adequately [19,20,21]. Cognitive distortions and affective deficits are part of the process that leads the offender to belittle the impact on the victim [22,23].

Sex offenders constitute a heterogeneous category that can be divided into different types (e.g., individuals who commit rape, individuals who molest children, female sexual assailants) according to the characteristics and motivations that move the person to commit the crime [24,25]. This heterogeneity can also be found in their personality profiles: multiple diagnoses or psychic characteristics linked to this behavior have been emphasized in literature [26,27,28,29,30,31]. The type of personality disorders most often found in the offenders include the narcissistic, antisocial, dependent, and psychopathic disorders [32]. The characteristics of the latter include the inability to establish full and mutually satisfying relationships with the others [33,34,35,36,37,38]. The classification of personality disorders does not help people to gain a complete understanding of the motivations at the root of the behaviors of sex offenders, and it does not give adequate information about the psychic dynamics underlying such behavior [39]. In addition to personality disorders, psychopathy is also a condition, or rather, a personality construct, which is particularly important to highlight due to the characteristics that accompany it and the high risk of recidivism that it may represent [40]. Psychopathy can be self-evident or (most of the time) associated with multiple forms of personality disorders sharing ego-syntonic types of behavior, characterized most often by a lack of a genuine feeling of guilt or remorse towards the others [41,42,43].

All that said, it seems necessary to be able to implement better assessment tools that offer the possibility of a specific description of a functioning profile of the individual perpetrator of sex crimes, taking into consideration as well the fact that, as shown by many international studies [44,45,46,47], the perpetrators of sexual abuse are more likely to repeatedly commit crimes (even if they are of a non-sexual type). The estimate of recidivism of the perpetrators of sexual abuse after four to five years is 10–15% compared to an estimated 8–9% of non-sexual violence, and approximately 40% of other types of recidivism [48,49].

The management of the inmate in the penitentiary environment is also very complex:

- In most of the cases they are first-time offenders who have not had any previous prison experience and who are condemned to long periods of imprisonment;
- It is difficult to guarantee adequate mental treatment interventions or suitable placement for these subjects within the prison facility;
- It is difficult to avoid the punishment dynamics that other prisoners impose upon them.

Unfortunately, therapeutic interventions and psychological treatments designed specifically for sex offenders are not widespread, despite the fact that at an international level, the literature indicates that the percentage of recidivism for untreated sex offenders is 17.3% compared to 51.3% of other offenses, a percentage that drops to 9.9% for the sex offenders who have had treatment for their sexual offenses, and 32.3% in case of non-sexual offenses [14].

Various treatment projects have been launched and experimented in some countries, such as the Challenge Project in England, the Naval Carnero Project in Spain, the Step Project, and the TBS system treatment in the Netherlands. These treatment projects are oriented to control perverse fantasies, a predictive element of the recurrence of abuse, with the aim of limiting deviant stimulation, and the identification of any condition of risk, feelings, moods, and thoughts that could lead to the recidivism of the behavior [42,50]. The *Sexual Offender Treatment Programme* (SOTP), offered to perpetrators condemned to imprisonment in Great Britain [51] (Middleton, 2006), Ireland, Finland, and Spain is included among the programs based on these principles [52] and it is particularly articulated in the various phases of treatment. In Italy we can only cite a few, limited experiences, such as the treatment project and model implemented at the Milan-Bollate Recruiting House (Lombardy Region/North-Italy) [53], as well as the Project of treatment and management of sex offenders on the Intense Treatment and Impairment Units promoted by the Italian Centre for the promotion of Mediation (also in Milan). Other interventions have been implemented in Puglia, in southern Italy (SPERARE) and in Tuscany, central Italy (For Wolf). Nevertheless, empirical research has not produced conclusive evidence yet of the therapeutic efficacy of prison treatment programs for sex offenders. The current treatment programs are characterized by many therapeutic goals and, as a result, they are not very integrated or standardized [54].

All that said, in this paper the authors show a pilot study performed in the prisons of central Italy in 2018, aimed at evaluating the feasibility of a more accurate delineation of the personality structures that characterize sex offenders, with a special focus on the traits that characterize the psychopathy construct, the description of the levels and modes of functioning of the individual, and the identification of risk factors, factors that could help to assess, in a concrete way, the risk of recidivism. Consequently, with the results of this evaluation one could be able to customize the treatments that can be implemented in prisons.

The authors can already anticipate that the obtained results can help expand research to a national level, a project which is currently underway.

## 2. Materials and Methods

The main research question is the following: Is it possible to assess the risk of recidivism of sex offenders through specific tools? In light of the results obtained from the administration of these specific instruments, what are the factors to be taken into greater consideration for the risk of recidivism?

An investigation will be carried out through a semi-structured interview aimed at assessing the criminological risk of social danger by means of the scoring of the Historical Risk Management-20-version3 (HCR-20 V3) and the Psychopathy Checklist-Revised (PCL-R), as well as the antisocial/psychopathic personality variables with the Personal Inventory dimensional (PID-5).

The research aims at the improvement of the evaluation and decision-making process of criminal justice operators with respect to the criminological and social risk associated with sex offenders.

Specifically, the authors expect that the results obtained through the administration of the instruments listed above will be able to increase in the operators the knowledge of risk factors for recidivism of sexual crimes so that they can intervene effectively.

The HCR-20 V3 is a tool not yet standardized in Italy. The authors considered it worthwhile to use this instrument in the study since it has into high capacity to predict the risk of recidivism. In fact, another objective of the study is to standardize the HCR-20 V3 in Italy as well.

Another goal is that upon the follow-up study one and two years after release, it can be empirically demonstrated that ex-prisoners assessed at risk of recidivism according to HCR-20 V3, PCL-R and PID-5, present a risk of committing new sex crimes significantly greater than other ex-offenders.

The last goal is that the analysis of the results offers useful support for implementing psychoeducational treatments specifically aimed at reducing risk factors for the recidivism of sexual crimes. In fact, the use of PID-5 makes it possible to provide essential information to the therapist involved in the treatment of sexual offenders. This can be possible since the static risk factors elicited using HCR20 v3, or the presence of a psychopathic style, cannot be modified. Thus, the therapist is able to work on the C-R factors of the HCR20 v3, following the facets and domains that are higher with respect to a medium score, with the aim of modifying the individual pathological traits and describing the risks of recidivism.

### 2.1. Recruitment of Sample

The study presented was reviewed and approved by the Ministry of Justice- Prison Administration Department (Ethics committee). The prisons that participated in the study were from central Italy (“Sollicciano” in Florence and “La Dogaia” in Prato).

The inmates who participated in the study exercised a voluntary choice.

The educators of the jails gave to researchers the list of inmates with the reported crimes. The researchers gathered them in a room in the ward and they explained the research project. Furthermore, everyone was explained that the data collected would be used only at a statistical level in the study and not individually. It was explained to inmates that the results would not be communicated to the management of the prison.

The recruitment of the subjects took place on a voluntary basis.

All of the participants were informed about the purpose of the study and a properly signed informed consent form was collected.

The necessary information about the confidentiality and anonymity of the data for privacy, obtained by replacing names with an identification code, have been given to the participants who were also informed of their right to withdraw from the research at any time and of the full voluntariness of the participation, the lack of any kind of compensation in case of participation.

The sample consists of a group of 44 subjects detained for sexual offenses with final conviction and a control group of 50 subjects detained for crimes against the non-sexual person. Sex offenders must be in the “end of sentence” condition of one or two years of detention so as to monitor any relapses after one/two years from release. Of the 44 sex offenders, 30 are involved in repeated sexual crimes.

Participants were told that they could leave the research at any time. In addition, participants were told that participation in the research will not bring any benefits.

### 2.2. Instruments

The most recent empirical results have shown how the assessment of the risk of recidivism requires valid and reliable instruments that are able to identify and discriminate those historical and actuarial factors that allow one to effectively estimate the likelihood of recurrence of the sexual crime [24,47,55].

Reducing recidivism rates requires more accurate definition of personality structures, with special focus on the traits that characterize the psychopathy construct; a description of the levels and modes of functioning of the individual that could help to direct and personalize the treatments that can be implemented inside the prison; and a risk and management assessment in order to plain the actions to implement to reduce risk and prevent recidivism and protect victims

For these reasons, the following tools were used:

- The personal data are collected on a survey form to detect social type variables capable of influencing the recidivism of sexual crimes, such as age, socio-economic status, educational qualifications, and other individual data.
- The HCR-20 V3 is a scale created in Canada by researchers at Simon Fraser University, aimed at assessing the risk of violence and recidivism. The literature data show that the HCR-20 is a valid and reliable test, capable of predicting the risk of future violence with a percentage of between 70% and 90%, as well as the outcome of treatment and any recidivism. It belongs to the third generation of the *Violence Risk Assessment* and follows the methods of *Structured Professional Judgment* (SPJ), in other words, a risk assessment based on a careful analysis of historical and actuarial data, but also on the clinical judgment of the expert, in order to make a credible forecast of the risk of violence. It is an instrument widely used in many countries for the assessment of the risk of violence in various fields [56].

It foresees 20 indicators arranged into 3 subscales, including 10 historical or the future risk and central to the treatment planning. The seriousness of the future risk is a judgment drawn by the interviewer from the results of historical and clinical factors and on the basis of the local presence of structures able to take care of the subject. Each item must be evaluated on a scale from 0 to 2 based on the degree of certainty of the presence of the risk factor. A score of 0 indicates that the factor is absent or that its presence is not supported by available information on the patient’s history; a score of 1 indicates that the factor is possibly or partially present, or that the available information indicates some evidence, although not definitive, of its presence; and a score of 2 indicates the absolute, clear, and certain presence of the considered factor.

For research purposes, considering the HCR-20 as a real statistical scale, we can sum up the score of the items that make up each of the three subscales so as to obtain partial scores related to the “weight” of the historical factors (where the score goes from 0 to 20), clinical and risk management (where the score goes from 0 to 10), and then add them together to get a total score (which goes from 0 to 40).

- The PCL-R enables the measurement of the psychopathy traits through 20 items, assessed on the basis of the data collected during the semi-structured interview and information collected from collateral sources [33]. It is aimed at the investigation of all of the areas of the life of the subject including infancy, affections, relationships, addiction, etc. Each item is evaluated on a Likert Scale at three levels: 0 when the trait is absent, 1 if it is partially present, or 2 when fully present. By applying the standard or pro-rating procedure, the PCL-R provides a dimensional assessment of the subjects in relation to their psychopathic characteristics. The higher the score, the greater the psychopathic characteristics. The normal population scores an average of approximately 8–10 points on the PCL-R, while the average criminals score an average of around 18–20 points [57]. The research takes into consideration subjects with a score ranging between 0 and 20 showing low psychopathy, those with a score of between 21 and 29 with average psychopathy (potentially psychopathic and worthy of a more in-depth evaluation), and finally subjects with a score of 30 or higher who have serious psychopathic traits and can therefore be diagnosed as psychopathic [34]. In a clinical environment such as that of the High Security Rampton Hospital in the United Kingdom, a score of 24 is defined as a cut-off for the placement of patients, perpetrators of crimes and positive to the PCL-R, in a specific section dedicated to psychopaths. There are two main sub-scales (Factor 1 “Interpersonal/Affective” and Factor 2 “Social Deviance”) consisting of “components” (“Interpersonal” and “Affective” for Factor 1, and “Lifestyle” and “Anti-sociality” for Factor 2). PCL-R demonstrated good internal consistency, good test-retested reliability, and a high degree of agreement among evaluators, even in different populations. There have already been significant correlations with Factor 2 “Social Deviance” and with Components 3 “Lifestyle” and 4 “Anti-sociality” [58].
- The PID- is a test that sheds light on the model of personality traits of the DSM-5 which has been developed directly by the American Psychiatric Association for the dimensional assessment of personalities [59]. It is a self-reporting evaluation of the personality consisting of 220 items. This instrument initially produces scores on 25 facets, or specific traits, which can be grouped together to specific domains of the pathological personality, including negative affectivity, detachment, antagonism, disinhibition, and psychoticism [60]. As such, this instrument may offer the chance to define more accurately the ways in which the person functions, highlighting in more detail any aspects of greater fragility and pathological functioning which it is necessary to intervene [32].

### 2.3. Data Analyses and Limits

All of the data collected were coded and inserted in a matrix in order to carry out descriptive analyses.

The data were entered with a database created with ACCESS 2007 (Microsoft, Redmond, Washington)).

The analyses were performed with the Excel program.

It is important to advance some critical considerations in this regard: the limited number of subjects examined in the sample does not allow to draw definitive and valid conclusions. Unfortunately, this is a fundamental problem for the limitations imposed by prisons on scientific research on account of the bureaucratic difficulties found in the access, and due to the fact that the sex offenders must be definitively condemned to take part in any research (sex offenders commit different categories of sexual crimes).

## 3. Results

### 3.1. Description of the Sample

The participants consisted of a group of 44 male offenders, aged between 25 and 81 years (M = 49.09; SD = ±13.59) who had been imprisoned for sexual offences.

Nationality: Italian (*n* = 42); Moroccan and Sri Lankan (both *n* = 1).

Civil status: divorced or separated (*n* = 31); unmarried (*n* = 7); married (*n* = 6).

Educational level: elementary school certificate (*n* = 1); secondary school diploma (*n* = 27); high school diploma (*n* = 16).

The majority of the sample (*n* = 32) had never attempted to undertake a study course to improve the educational level already achieved, while of the 12 participants who began a study course, 10 abandoned it soon.

Occupation: the whole sample declared to be blue-collar workers.

Family of origin: normal composition (*n* = 38); death, separation/divorce, abandonment (*n* = 6).

Abuse experiences: physical/psychological abuse in childhood (*n* = 30); sexual abuse (*n* = 0); use/abuse of alcohol and drugs (*n* = 26); use/abuse of alcohol (*n* = 6).

Victims of sex offenders: in the majority of cases the victims were underage (*n* = 25).

Recidivism: *n* = 38.

### 3.2. Results of the HCR-20V3

The average score for the HCR-20 V3 sample is 21.40 with SD = ±1.55.

Regarding the H factors, the total average of the sample is 13.86, SD = ±2.14. This means that historical factors play an important role in predicting the risk of future violent behavior.

In particular, the H1 factor resulted interesting since the whole sample presents a history of problems related to violence committed in adolescence. The episodes are serious, repeated over time, and constant.

As regards the C clinical factors, the total sample average is 6.58 SD = ±1.22 and as a result these factors, similar to the historical factors, are also significant in predicting the risk of recidivism.

In particular, the C1 factor resulted interesting and in fact, the whole sample presents significant problems of insight, especially concerning the risk of violence for which the subject has no awareness, understanding or knowledge of its functioning in relation to violence or factors and processes that put them at the risk of violence.

Below, Table 1 shows the items resulted particularly significant. Most of the sample had a childhood characterized by traumatic events, an adolescence characterized by intense violence, and had problems with alcohol and drug abuse. Regarding clinical factors, the sample has a lack of insight. It is important to know what elements sex offenders have in common in order to intervene on them and carry out effective treatment programs.

### 3.3. Results of the PCL-R

The total score of the PCL-R resulted in an average of 16.47 (*T* = 43; SD = ±8.30).

*N* = 28 present a low level of psychopathy with total values ranging between 3 and 17, with an average score of 11.25 and SD = ±1.99; *n* = 3 turn out to have a medium level of psychopathy (21 ≤ X ≥ 29) and *n* = 5 are collocated at the high level of psychopathy (X ≥ 30). Table 2 shows the results of the PCL.R.

### 3.4. Results of the PID-5

The analysis of the results of the PID-5 Personality Inventory was carried out according to the test manual on the average of the scores obtained in the individual facets and domains.

The PID-5 is especially important in the identification of the personality traits useful for the definition of an alternative to the personality disorders model (Section III of the DSM-5) as well as monitoring the effects of the psychotherapeutic intervention. In fact, their use is recommended “in *the research and evaluation phase as instruments that can help the clinician in the decision-making process and not just as the sole support for making a clinical diagnosis*”. In the absence of a codified and commonly accepted cut-off point [61], a score higher than 1.00 has been considered positive, and capable of defining an anomalous or pathological functioning that “makes it possible for the clinician to consider the dysfunctions of the subject’s personality”.

We considered the facets as positive when more than 50% of the subjects had a score higher than 1.00: separation anxiety (51.22%), anxiety (63.41%), emotional lability (60.98%), rigid perfectionism (75.61%), perseveration (60.96%), and suspiciousness (58.54%). It must be emphasized how the Impulsivity facet, which is specifically related to the implementation of violent behavior [60] reaches just the limit value (48.78%). Regarding the domains (main value of the averages of several facets), the average value of 1.00 in at least 50% of the subjects is obtained for the negative affectivity (56.10%) which combines the averages of anxiety, emotional lability, and separation anxiety.

## 4. Discussion

Despite the limits of the study (limited number of subjects examined, different categories of sexual crimes) we are able to confirm some elements, already confirmed in the literature, and highlight new areas for research and investigation.

Based on the results that have emerged, from the combination of the administered three tests (HCR20v3, PCL-R, PID-5), several traits have been revealed that can direct the interventions aimed at mitigating the risk of recidivism, during the imprisonment phase, and even more so during the return to the individual’s social context.

From the data retrieved from the HCR20v3, in the assessment of the risk of violence, a marked positivity emerges in the factors of the sub-scale H, consisting in anamnestic-static factors, and more specifically those related to:

- The use of alcohol and/or drugs;
- The early onset of episodes of violence at a young age, prior to the offence committed;
- A family history characterized by violence suffered or witnessed by the subject and frequent negligence;
- Difficulty in intimate relationships and often also in friendship relationships;
- The presence of a personality disorder of the B cluster.

Clinical factors point to a positivity due to the lack of insight, the persistence of violent ideation, and behavioral and affective instability.

As for the R factors, the lack of specialized local services for the treatment and assistance to perpetrators of sexual violence arises as a general problem. This makes it difficult to continue the treatment, started inside the prison, outside.

In general, we can say that the risk factors such as an early school dropout, a dysfunctional family history in which the individual faces traumatic experiences or situations of institutionalization during childhood and adolescence, the use/abuse of drugs, and previously committed crimes seem to be factors that strongly influence the development of psychopathy and, therefore, the actual risk of a repeat offense after the release.

In the detection of a condition of psychopathy, the PCL-R, which is considered the gold standard, proved to be positive only in very few cases. This result could depend on the difficulty in the collection of adequate information, which is not just clinical, for the interviewed subject’s past and considering that even the prison officials find often difficult to gather information.

With regard to the average scores of the four components, we can observe (Table 2): average scores lower than the normative data for the Component 1 “Interpersonal”; average scores slightly above the normative data for the Component 2 “Affective” and Component 3 “Life Style”. Finally, average scores in line with normative data for the Component 4 “Antisociality”.

With regard to the average scores of “Factors”, we can observe that Factor 1 “Interpersonal/Affective” is lower than the normative data, and Factor 2 “Social Deviance” is slightly above the normative data.

The influence of evolutionary components appears to be in line with the theory of psychopathy as a deviant developmental disorder, reinforcing the need for early and lasting interventions in families identified as being at risk by the deputed agencies. The family remains the place where the child creates, researches, and strengthens his defenses. In this sense, a family that fails to convey to the child a sense of security is confirmed to be a very important risk factor. The use/abuse of substances have an influence on the typical interpersonal traits of the psychopath. Early school dropout, notoriously an element that affects cognitive and experiential maturation, seems to play a decisive role in the psychopathic aspects of Component 3 “Lifestyle” of PCL-R and Factor 2 “Social deviance” of PCL-R. An early abandonment of school education jeopardizes the achievement of critical thinking and affective maturation appropriate to information processing.

They can therefore develop cognitive distortions in the world around them, especially in relation to sexuality.

In the end, we can say (see results of PID-5) that the two main dimensions where the results emerged are: the affective dimension characterized by anxious symptomatology, lability of emotional control, and the inability to handle a relationship; and the ideational dimension, which is characterized by rigid and interpretative modalities, with relational and communicative models that are rigid and negate/neglect.

On the whole, we can state that the sex offenders show a functioning profile of the ego characterized by two aspects: a problematic affective dimension, since it combines characteristics of anxiety and separation anxiety, activated by the inability to accept the ending of a relationship, a lack of control over emotions that appear to be characterized by instability, a tendency for exhaustion, and inconstancy; and the association between rigid perfectionism, perseveration, and suspiciousness, a constellation of characteristics that makes these subjects very close to a “paranoid” and gives the rise to the re-proposing of rigid and immutable relational and communicative models in which they are unable to accept other points of view despite, or perhaps above all, the search for an autarchic perfection that does not tolerate any diversity of opinions or choices in the field of emotional relationships.

## 5. Conclusions

The results of the study identify the importance of working on the contextual factors of the participant (both the family system and the psychological development aspects) in all those situations that have been identified as being at risk for the onset of a criminal conduct.

It is desirable to create social projects aimed at strengthening the nurturing factors of families, and especially of high-risk individuals. Indeed, we have seen how historical factors play a very important role in predicting the future behavior of the subject.

Finally, the authors here believe that the results obtained provide further knowledge about the main and dominant personality traits in sex offenders and the risk factors of sex abuse for which it is necessary to intervene individually to prevent recidivism.

Nevertheless, it is important to advance some critical considerations in this regard:

- The limited number of subjects in the sample examined does not allow for drawing definitive and valid conclusions;
- The sex offenders in Italian penitentiary facilities commit different categories of crime;
- An increase in the number of evaluations would make it possible to conduct an analysis on several sub-categories with respect to the crimes and personality characteristics;
- The PID-5, although widely used, should always be tested with the validity scales currently in the experimentation phase in order to identify the subjects that give random responses [32];
- The success of individualized programs should be evaluated in an attempt to prevent the risk of recidivism.

The authors, also believe that the success of individualized programs should be evaluated in an attempt to prevent the risk of recidivism. For this reason, the authors are starting to implement targeted interventions on this category of perpetrators. This is possible thanks also to the support of Contrasti Association, founded in 2017, which pursues as its main objectives the sensitization of institutions to a clinical-criminological approach to the issue of sexual aggression. The treatment is necessary for a real effectiveness of the punishment with respect to the prevention of the risk of recidivism, since the rehabilitative function of the punishment is complementary to that of retribution; dissemination of a culture of treatment as a more effective tool for the prevention and rehabilitation of the sexual offender and for the protection of society; building and maintaining contacts with international associations aimed at research and treatment of the sexual offender.

## Figures and Tables

**Table 1:** HCR20v3 items resulted significant.

**H**	***n***	**Explanation**
H1 “Past Violence”:	44	history of problems related to violence committed from age 13 onwards. The episodes are serious, repeated over time and constant.
H3 “Relational instability”	36	considerable problems in initiating and maintaining intimate relationships.
	8	problems also arise in non-intimate relationships followed by socially isolation, violence or experience of inappropriate sexuality.
H5 “Problems of Drugs use”	39	previous history of use/abuse of alcohol and drugs.
H7 “Presence of Personality Diseases”	40	antisocial, psychopathic or dissocial personality disorder. This emerges as a symptom of a history of serious problems resulting from rigid or maladjusted personality traits related to the interpersonal style, behavioral control, emotionalism.
H8 “Early traumatic experiences”:	30	very adverse experiences in childcare that may have interrupted a regulatory development, attachment processes, or the learning of pro-social attitudes and problem-solving skills More specifically, *n* = 23 report a very traumatic childhood history with episodes of parental violence and abuse.
H9 “Violent attitudes”	41	previous problems involving violent behavior and support of violence.
**C**	***n***	**Explanation**
C1 “Insight”	44	significant problems of insight, especially concerning the risk of violence which the subject has no awareness of, understanding or knowledge of its functioning in relation to violence or factors and processes that put them at risk of violence.
C4 “Impulsiveness”	41	serious problems regarding the maintaining of adaptive behaviors on the emotional and behavioral level
**R**	***n***	**Explanation**
Risk management	36	future problems are evident in relation to the implementation of general action plans that resort to an appropriate use of services or professional programs.
	41	problems in the individual assistance since there is no actual social network, moreover the emotional support is inadequate, there are no clear or well-organized plans for intervention, the social network consists of subjects who could have a negative influence, and help and assistance are inadequate to fit their daily needs and activities.

*n* = 44.

**Table 2:** Results of PCL-R.

**Components**	**M**	**SD**	**T Score**
1: Interpersonal	3.6	±3.01	46
2: Affective	5.30	±2.99	51
3: Life Style	6.30	±0.96	51
4: Antisociality	4.21	±1.22	50
**Factors**	**M**	**SD**	**T** **Score**
1: Interpersonal-Affective	8.55	±2.69	47
2: Social Deviance	10.9	±2.25	52

*N* = 44.

## Data Availability

Data available on request due to restrictions eg privacy or ethical The data presented in this study are available on request from the corresponding author. The data are not publicly available due to because the creation of a publicly accessible database was not planned.
